# Low Back Pain and Foot Drop Associated with Dog Tapeworm Infection

**DOI:** 10.4269/ajtmh.20-1011

**Published:** 2021-01

**Authors:** Ayush Agarwal, Venugopalan Y. Vishnu, Ajay Garg

**Affiliations:** 1 Department of Neurology, AIIMS, New Delhi, India;; 2 Department of Neuroradiology, AIIMS, New Delhi, India

**Figure 1.**
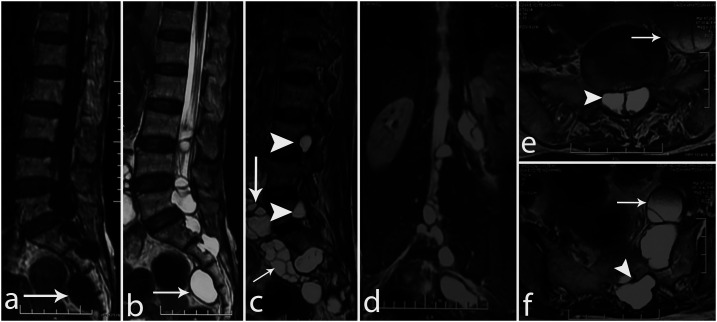
Sagittal T1-WI (**A**), sagittal (**B** and **C**) and coronal T2-WIs (**D**) show multiple well-defined cystic lesion in the intraspinal, preverebral, and presacral spaces (arrows in **B** and **C**) and in neural foramina (arrowsheads in **C**). Like the CSF, the lesions are hypointense on T1-WI (**A**) and hyperintense on T2-WI (**B** and **C**). Axial T2-WIs at L5 (**E**) and S1 levels (**F**) show intraspinal lesions (arrowheads in **E**) and lesions invoking the left psoas muscle (arrows in **E** and **F**).

A 61-year-old woman presented with low back pain associated with radiation to left lower limb since the past 1 year, with left foot drop since the past 1 month. Examination revealed wasting of the left lower limb with a foot drop. The remaining neurological examination was normal. Magnetic resonance imaging of the spine revealed diffusely bulky and multi-septated homogenous fluid-filled lesions (with signal intensity similar to cerebrospinal fluid [CSF]) in the intraspinal compartment from L3 to sacral vertebrae, causing extradural compression, and prevertebral and paravertebral locations (Figure 1). Contrast-enhanced computed tomography of the abdomen revealed similar cysts in the retroperitoneum, liver, and left psoas muscle. Serum ELISA was positive for *Echinococcus* IgG antibody (value-19.31; normal < 9), thus confirming the diagnosis of disseminated hydatidosis.

Hydatid disease is a parasitic infection caused by the larval form of *Echinococcus granulosus*.^1^ Humans are intermediate hosts who become infected by accidental consumption of infected food/water,^2^ with liver and lungs being the commonest sites of involvement.^3^ Bone involvement is rare, occurring in less than 2% cases, with spinal involvement occurring in half of those cases.^3^ The thoracolumbar spine is the common site of involvement, with involvement of the sacral spine being very rare.^4^ ELISA serology has a sensitivity of 80–100% for hepatic infections but only 25–56% for other organ involvement.^5^

In patients hailing from endemic regions and presenting with chronic low backache, with imaging suggestive of space-occupying lesions, hydatid disease should be considered in differential diagnosis. Early diagnosis and treatment lead to good clinical outcomes.
